# Combating Drug-Resistant Bacteria Using Photothermally Active Nanomaterials: A Perspective Review

**DOI:** 10.3389/fmicb.2021.747019

**Published:** 2021-11-12

**Authors:** Kawaljeet Kaur, Sagar Reddy, Pramod Barathe, Varsha Shriram, Uttpal Anand, Jarosław Proćków, Vinay Kumar

**Affiliations:** ^1^Department of Biotechnology, Modern College of Arts, Science and Commerce, Ganeshkhind, Savitribai Phule Pune University, Pune, India; ^2^Department of Botany, Prof. Ramkrishna More College, Savitribai Phule Pune University, Pune, India; ^3^Department of Life Sciences, Ben-Gurion University of the Negev, Beer Sheva, Israel; ^4^Department of Plant Biology, Institute of Environmental Biology, Wrocław University of Environmental and Life Sciences, Wrocław, Poland

**Keywords:** antibiotics, multidrug resistance, nanoparticles, drug efflux pumps, ROS, antimicrobial resistance, photothermal agents, photothermal therapy 3

## Abstract

Injudicious use of antibiotics has been the main driver of severe bacterial non-susceptibility to commonly available antibiotics (known as drug resistance or antimicrobial resistance), a global threat to human health and healthcare. There is an increase in the incidence and levels of resistance to antibacterial drugs not only in nosocomial settings but also in community ones. The drying pipeline of new and effective antibiotics has further worsened the situation and is leading to a potentially “post-antibiotic era.” This requires novel and effective therapies and therapeutic agents for combating drug-resistant pathogenic microbes. Nanomaterials are emerging as potent antimicrobial agents with both bactericidal and potentiating effects reported against drug-resistant microbes. Among them, the photothermally active nanomaterials (PANs) are gaining attention for their broad-spectrum antibacterial potencies driven mainly by the photothermal effect, which is characterized by the conversion of absorbed photon energy into heat energy by the PANs. The current review capitalizes on the importance of using PANs as an effective approach for overcoming bacterial resistance to drugs. Various PANs leveraging broad-spectrum therapeutic antibacterial (both bactericidal and synergistic) potentials against drug-resistant pathogens have been discussed. The review also provides deeper mechanistic insights into the mechanisms of the action of PANs against a variety of drug-resistant pathogens with a critical evaluation of efflux pumps, cell membrane permeability, biofilm, and quorum sensing inhibition. We also discuss the use of PANs as drug carriers. This review also discusses possible cytotoxicities related to the therapeutic use of PANs and effective strategies to overcome this. Recent developments, success stories, challenges, and prospects are also presented.

## Introduction

Recent years have witnessed a sharp increase in the incidence and magnitude of bacterial drug resistance [also known as antibiotic resistance (ABR), or antimicrobial resistance (AMR)] (Khare et al., 2021a, b; Tiwari et al., 2021). Initially considered largely a nosocomial problem, bacterial drug resistance has now reached community environments (Shriram et al., 2018; Anand et al., 2019; Friedrich, 2019; Taneja and Sharma, 2019; Khare et al., 2021a, b; Tiwari et al., 2021). The global rise of bacterial drug resistance poses serious threats to human health and the environment. Drug resistance is the ability of bacterial pathogens to survive the drugs/antibiotics to which they are exposed (Anand et al., 2020). The World Health Organization (WHO) has declared bacterial drug resistance a global menace that undermines the practice of medicines (Morrison and Zembower, 2020). The statistics on drug-resistant infections project around 10 million deaths per year by 2050, with Asia and Africa accounting for 4.7 million and 4.1 million deaths, respectively (O’Neill, 2014).

Inappropriate and/or injudicious use of conventional antibiotics is reported to be the main driving force behind the increasing resistance to drugs (Tiwari et al., 2021) and the condition is worsening further due to the drying pipeline of new and effective antibiotics (Khare et al., 2021b). There are reports of increasing levels of bacterial drug resistance, ranging from multidrug resistance (MDR) (acquisition of non-susceptibility to at least one agent in 3 or more antibiotic classes) to extensive drug resistance (XDR) (non-susceptibility to at least one agent in all but 2 or fewer antibiotic classes) and pandrug resistance (PDR) (non-susceptibility to all antibiotic classes) (Exner et al., 2017; Spengler et al., 2017; Shriram et al., 2018; Koch et al., 2021). Since the first reports of drug resistance on *Enterobacteria* in the 1950s, numerous bacterial strains with drug-resistant phenotypes have been reported, and this number of strains as well as their abilities to resist antibiotics are ever increasing. The increase in frequency, magnitude, spread, and persistence of drug-resistant bacterial phenotypes has contributed significantly to the challenge. The emergence of new resistance mechanisms (along with known drug exclusion/resistance mechanisms) in these bacterial strains contribute to the inefficacy of existing drugs, prolonged illness, and increased expenditures (WHO, 2020). This situation demands newer strategies for the eradication of drug-resistant bacteria, including new and effective antibacterial agents with potential clinical applications. Although there are some successful events to identify potent antibacterial agents, for example metal/metal oxide nanomaterials, their translational success is poor (Mapara et al., 2015; Khare et al., 2021a). Main challenges for limited translation to clinic include poor data availability on interactions with human cells, tissues and organs, optimal dose, recognition of appropriate routes and possible toxicities and side effects following acute and long-term exposure (Lee et al., 2019).

Over the past few decades, several reports have highlighted the effective use of nanomaterials as potent antibacterial agents, including inorganic and organic nanomaterials. Among them, metal nanoparticles, in particular silver (Ag-NPs) and gold nanoparticles (Au-NPs) are studied more extensively to assess their bactericidal activity (Mapara et al., 2015), as well as drug resistance reversal activities (Hemeg, 2017; Lee et al., 2019; Khare et al., 2021b). However, the less stable (Levard et al., 2012) and toxic nature (Sahu and Hayes, 2017) of these NPs have limited their clinical application. Overall, multifactorial resistance against such agents offered by bacterial pathogens, in addition to the toxicity issues and potential side effects along with their stability and drug-releasing issues in dynamic gut environment, are limiting the clinical applications of potent, conventional antibacterial nanomaterials. Liposomes were seen as attractive additional or alternative antibacterial drugs and/or drug carriers. However, issues related to the use of liposomes have limited their clinical uses, including physical and chemical instability issues, the thermolabile nature of liposomes, in addition to other issues such as low loading capacity (Eleraky et al., 2020).

In recent years, photothermal therapy (PTT) has attracted attention from the scientific community and is emerging as a potent (Guo et al., 2020), non-invasive approach for combating pathogens, including drug resistant ones (Yang et al., 2021). PTT involves the exploration of photothermally active nanomaterials (PANs) including gold, carbon, iron oxide (FeO), etc. that exhibit high absorption in the near-infrared (NIR) region of the electromagnetic spectrum (Bao et al., 2016; Li X. et al., 2020). The mechanism of action lies in the ability of PANs to absorb NIR light and its rapid conversion into heat, thereby increasing the surrounding temperature of the irradiated organism (Borzenkov et al., 2020). However, there are some limitations, which need attention before full-potential exploration of PTTs and PANs, in particular, as therapeutic antibacterial agents effective against drug-resistant pathogens.

In this review, we present an account of recent developments in the identification of potent PANs and the exploration of PAN-based PTTs to combat drug-resistant pathogens by targeting their main drug-resistant determinants. In addition, the use of PANs as drug carriers has also been discussed. This review also discusses the possible cytotoxicities related to the therapeutic use of PANs and effective strategies to overcome them. Contemporary developments, major success stories, challenges, and prospects are also presented.

## Photothermally Active Nanomaterials as Potent Antibacterial Agents

Photothermally active nanomaterials are known to exhibit broad-spectrum antibacterial effects driven mainly by the photothermal effect characterized by the conversion of absorbed photon energy into heat energy (Borzenkov et al., 2020). PANs exhibit these photothermal properties because of the d-d band energy transitions and surface plasmons (surface electron resonance) referred to as excitation of electrons by incident light photons. They are highly dependent on the concentration of nanoparticles, wavelength, conversion efficiency, irradiation intensity, morphology, and the type of nanomaterial used (Borzenkov et al., 2020). As the properties of plasmonic nanoparticles that exhibit surface electron resonance strongly depend on the synthesis process and the surrounding environment of the nanoparticles, the properties of non-plasmonic nanoparticles depend less on the dimensions and shape (Borzenkov et al., 2019). Applications of PANs have been reported in the field of chemotherapy, cancer treatment, and photodynamic therapeutics (Hu et al., 2018; Yang et al., 2019).

There are reports on organic and inorganic PANs constituted with various metals, carbon, polymers, noble gases, metal oxides, and nanoscale metals targeting bacterial pathogens, including drug-resistant phenotypes (Feng et al., 2017). Approaches including PTT and photodynamic therapy (PDT) exploring the PANs are gaining momentum for the treatment of bacterial infections. PDT is the non-invasive therapeutic approach for bacterial infection treatments that uses low-power laser radiations, singlet oxygen, and photosensitizer for bacterial ablation (Rajesh et al., 2011). However, PDTs are finding their limited applications mainly due to their low delivery efficiency, while PTTs are gaining momentum as potent agents for containing bacterial infections (Chen et al., 2020). De Aberasturi et al. (2015) advocated various applications of these plasmonic nanomaterials in PTT and PDT. In PTT, photothermal agents (PTAs) transform light energy into thermal energy, and the generated heat triggers the rupturing of cell membranes, denaturation of proteins, and ablation of bacteria. Researchers have identified different types of PTAs with organic/inorganic compounds, metals, and carbon-based nanomaterials (Bao et al., 2016). Their easy synthesis, rapid body clearance after use, photostability, and high photothermal conversion efficiency make PTAs effective antibacterial agents with potential for therapeutic applications (Chen et al., 2020). Furthermore, an exhibition of extraordinary light-matter interactions, plasmonic properties, free electron pairs, energy bandgap, optical conductivity, optical wavelength, conductance, and morphological characteristics of nanomaterials are also considered essential factors of PTAs in the PTT (Liu S. et al., 2020). PANs are used as antibacterial agents by irradiation of light ranging from invisible NIR spectra with 650–900 nm wavelength to the ultraviolet (UV) spectrum, resulting in thermal relaxation and increased temperature (Borzenkov et al., 2020). Due to the easy operational, safe profiling, and deep tissue penetration capabilities, NIR light is preferred in PTTs.

Various PANs such as zinc-based nanomaterials (Li et al., 2019a), silver-based nanomaterials (Zhu et al., 2020), gold-based nanomaterials (Li X. et al., 2020), metal organic frameworks (MOFs) (Luo et al., 2021), copper-based nanomaterials (Han et al., 2021), and graphene-based nanomaterials (Lv et al., 2021) exhibiting photothermal activity have been investigated to combat drug resistant bacteria (Ren et al., 2020). Due to their structural compositions and chemical nature, MOFs are considered the front-runner antibacterial PANs (Zheng et al., 2021). Han et al. (2020a) developed copper-doped MOFs possessing enhanced photocatalytic activity and photothermal effects to treat *Staphylococcus aureus*-infected wounds under 660-nm light irradiation, which resulted in striking antibacterial efficiency (99.7%). Silver phosphate (Ag_3_PO_4_) nanoparticles, decorated with defective titanium oxide in black urchin shape (TiO_2_) (BU-TiO_2_-X/Ag_3_PO_4_) in the nanospike shape, showed high antibacterial efficiency against *Escherichia coli* (99.76%) and *S. aureus* (99.85%) under light irradiation (Xu et al., 2021). Similarly, zinc oxide carbon dots modified graphite carbon nitride nanocomposites (ZnO/CDots/g-C3N4) showed high antibacterial efficiency against *S. aureus* (99.97%) and *E. coli* (99.99%) under visible light irradiation (Xiang et al., 2020). Furthermore, *in vivo* clinical studies in mice wounds infected with *S. aureus* revealed the wound healing abilities of nanocomposites.

## Targeting Major Drug Resistance Determinants in Bacterial Pathogens With Photothermally Active Nanomaterials

**FIGURE 1:**
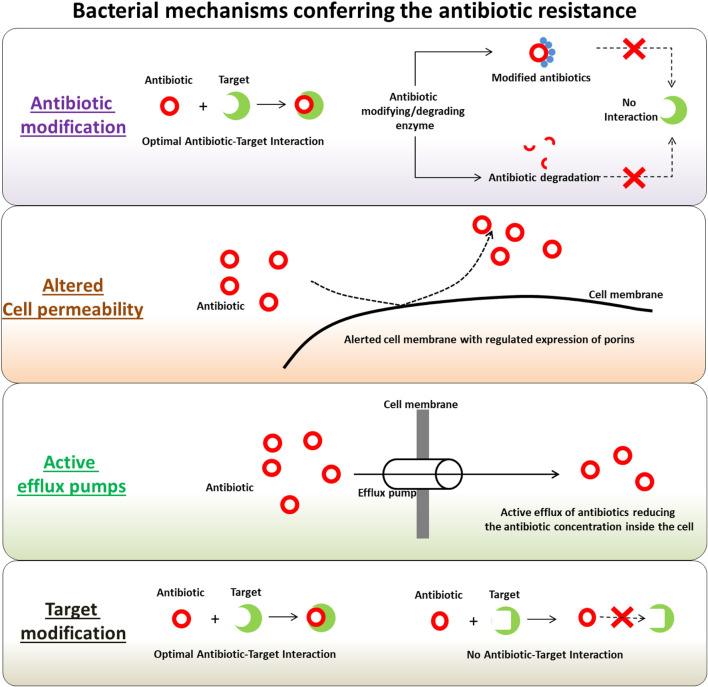
Major bacterial drug resistance mechanisms. Adapted from Khare et al. (2021a). Copyright (2021) Frontiers Media.

Bacterial pathogens, especially MDR or XDR strains, have a highly active self-defense armory that helps them survive against antimicrobial drugs, antibiotics, and pesticides (Khare et al., 2021a). These mechanisms often work in tandem, ensuring bacterial protection against wide-spectrum drugs, and thus driving them into “superbugs.” The main determinants of drug resistance include cell permeability and drug efflux pumps, the ability of these strains to modify or destroy applied antibiotics, and the modification of antibiotic targets (Figure 1). In particular, PANs have been characterized as striking antibacterial agents with the potential to combat drug-resistant strains, as they have been reported to target the major drug-resistant determinants of these bacterial pathogens (Table 1).

**TABLE 1:** Photothermally active nanomaterials against bacteria with targeted drug-resistant determinants.

Nanomaterial	Parameters			Targeted drug-resistance determinant	Mode of action	References
		
	Light	Bacterial pathogens and disinfectant intensity	Nanomaterial concentration			
TiO_2_ nanotubes coated with MoS_2_ nanoflowers (TiO_2_NTs@MoS_2_)	Visible light irradiation	Drug-resistant, extended spectrum β-lactamases (ESBL) producing *Escherichia coli* and methicillin resistant *Staphylococcus aureus*	–	Membrane permeability	Visible light-induced hyperthermia, ROS production (O_2_^•–^)	Lin et al., 2021
Cu nanoparticle-decorated two-dimensional carbon nanosheets (2D C/Cu)	Near-infrared light	Methicillin resistant *S. aureus* (99.97% ± 0.01%), *E. coli* (99.97% ± 0.01%) and *S. aureus* (99.96% ± 0.01%)	200 ppm	Membrane permeability	Increase in cell permeability, permeation of released ions, cytoplasm leakage, disruption of intracellular metabolic pathways, loss of cellular homeostasis and permanent damage	Song et al., 2021
Gold silver nanocages (GSNCs)	Near-infrared light	Multidrug resistant *E. coli* (95%) and methicillin-resistant *S. aureus* (98%)	32 μg/mL	Biofilm formation	Release of silver ions, upregulation of intracellular ROS, increase in intracellular oxidative stress, and bacterial damage.	Qin et al., 2021
Ciprofloxacin-conjugated gold nanorods (P(Cip-b-CB)-AuNRs)	Near-infrared light	Methicillin-resistant *S. aureus* (92.3%)	125 μg Au/mL	Membrane permeability	Cell membrane disruption via PTT, improving permeability and antibiotic sensitivity, leading to lipase-triggered ciprofloxacin release at the site of bacterial infection and their biofilms	Yin et al., 2021
Chitosan (CS) encapsulated multifunctional metal-organic nanoparticles (Fe-TCPP@CS NPs)	White light and laser irradiation	Methicillin-resistant *S. aureus* (97%) and *E. coli* (98%)	100 and 20 μg/mL, respectively	Membrane permeability	Rapid production of intensive ROS (^1^O_2_) and electrostatic binding of the nanoparticle with the cell membrane resulting in an improved antimicrobial effect	Zhang et al., 2020
Phospholipid-decorated gold nanorods (DSPE-AuNR)	Laser beam excitation	*Pseudomonas aeruginosa* (99.998%)	0.125–0.03 nM	Biofilm formation	Photothermolysis of bacteria in planktonic or biofilm cultures	Al-Bakri and Mahmoud, 2019
Hemoglobin-functionalized copper ferrite nanoparticles (Hb-CFNPs)	Near infrared light	*E. coli* (100%) and *S. aureus* (96.4%)	20 μg/mL	Membrane permeability	Hydroxyl radical production	Liu et al., 2019
Silver triangular nanoparticles (Tri-Ag)	Near-infrared laser light	Penicillin-resistant ESBL *E. coli* (87%) and methicillin-resistant *S. aureus* (90.1%)	67.4 μg/mL	Membrane permeability	Release of silver ions causing perforation and holes in the cell membrane	Qiao et al., 2018
Polydopamine-assisted Au-hydroxyapatite nanorods (PDA@Au-Hap NRs)	Near-infrared light	*E. coli* (96.8%) and *S. aureus* (95.2%)	200 μg/mL	Membrane permeability	Membrane integrity disruption via hydroxyl radical production	Xu et al., 2018
Polyethylene glycol-functionalized molybdenum disulfide nanoflowers (PEG-MoS_2_ NFs)	Near-infrared light	Ampicillin-resistant *E. coli* (89%) and endospore-forming *Bacillus subtilis*	150 μg/mL	Membrane permeability	Induced hyperthermia, hydroxyl radicals disrupting integrity of bacterial cell membranes	Yin et al., 2016

Several recent reports are coming on organic and inorganic nanomaterials for their photothermal ablation/destruction of drug-resistant bacteria (Zhao et al., 2021). Various metal oxides, non-metal oxides, and semiconductor-based nanomaterials have been investigated for their potential photothermal and photocatalytic activity against drug-resistant bacteria that possess different inhibition mechanisms such as radical formation, efflux, and biofilm inhibition for the killing of bacteria and are discussed below.

### Antibacterial Activities of Photothermally Active Nanomaterials via Reactive Oxygen Species Generation

Photothermally active nanomaterials are known to target and destroy/inactivate bacterial cell membranes, the first and most powerful line of bacterial defense, by decreasing cell permeability and the generation of reactive oxygen species (ROS) (Agnihotri et al., 2020). Singlet oxygen (^1^O_2_), electron (e^–^), hydroxyl radicals (•OH), and superoxide radicals (O_2_^•–^) have been reported to be directly involved in the loosening of permeability of the microbial membrane and the ultimate death of the target microorganism (Mapara et al., 2015; Feng et al., 2017). These radicals formed can cause damage to the cell membrane through lipid peroxidation, leading to the destruction of organelles and the ultimate death of microbes. Under oxygen deprivation conditions such as anaerobic environments, bacterial cells behave as electron acceptors, where direct entry of electrons leads to cell death. Several nanomaterials such as graphene oxide (GO) (Linklater et al., 2018), copper oxide (CuO) (Kung et al., 2017), zinc oxide (ZnO) (Patra et al., 2015), and titanium dioxide (TiO_2_) (Ranjan and Ramalingam, 2016) have been used for the inactivation of bacteria as cell permeabilizers. GO nanomaterials with sharp edges as unique property are known for the ablation of drug-resistant bacteria by causing piercing in membranes along with oxidative stress induction (Menazea and Ahmed, 2020). The interaction between GO and the bacterial membrane increases the microbial acquisition and can be used as PTAs attributable to greater heat transfer ability, where the photothermal effect of GO can be activated using the NIR and visible spectrum (Vangara et al., 2018). Europium-vancomycin-functionalized reduced-GO (Eu-Van-rGO), responsive to NIR light, were reported for their bactericidal properties against MDR strains through a heat generation mechanism (Yang et al., 2014). Furthermore, Li X. et al. (2020) reported highly effective photoactive gold nanoclusters decorated with amine-functionalized GO (Au-GO-NH_2_) nanosheets that photothermally inactivated bacteria by capturing them, generating heat, and producing ROS.

In addition to the bactericidal activities, there are also reports on the synergetic effects of nanomaterials and antibiotics against drug-resistant pathogens. The synergetic effect of reduced GO and gold nanostar, known as rGO/AuNS nanocomposites under 808 nm NIR irradiation, resulted in increased photothermal and antibacterial activity through membrane disruption and hyperthermal effect, leading to complete death of methicillin-resistant *S. aureus* (MRSA) (Feng et al., 2019). Similarly, the combination of Ag-NPs with GO the rGO/Ag nanocomposites under NIR irradiation showed enhanced photothermal activity leading to effective bactericidal potencies by disrupting the microbial membrane and generating ROS in *Klebsiella pneumoniae* and *E. coli* (Tan et al., 2020). Recent reports on oxygen-deficient Ni/reduced graphene oxide (Ni/rGO) nanocomposites demonstrated enhanced photothermal and photocatalytic activity under NIR irradiation by increasing GSH loss and destroying the microbial cell membrane through the formation of O_2_^•–^ and •OH radicals (Zhang Z. et al., 2021). The synergistic effects of GO with azithromycin antibiotics such as modified polyethyleneimine-citraconic anhydride (PEI-CA) and azithromycin-loaded GO nanosheets (AZI@GO-PEI-CA) as antimicrobial agents have been studied where PEI-CA improved membrane anchoring and AZI-GO resulted in inhibition of membrane piercing, protein and ribosome synthesis in *S. aureus* and *E. coli* strains, presenting them an intriguing tool for microbial infection abatement (Huo et al., 2021). The use of human serum albumin (HSA)/rGO/*Cladophora glomerata* (Cladophoraceae) bionanocomposites as a PTA was also identified with antimicrobial activity under sunlight/NIR irradiation mediated by ROS generation (Amina et al., 2021). Similar results were observed using graphitic carbon nitride-based nanomaterials (g-C_3_N_4_) with photocatalytic antibacterial activity (Kong et al., 2021). Because of environmental friendliness and inexpensive production costs, ZnO-based nanomaterials have been shown to be exceptionally appealing for microbial photoinactivation via NIR and UV radiation. ZnO nanoparticles are considered to be primarily involved in bacterial inactivation by generating ROS and inducing apoptosis (Mishra et al., 2017).

In addition to ROS generation, other mechanisms such as controlled release of Zn^2+^ ions from ZnO nanoparticles also play an important role in bacterial killing. Liu Z. et al. (2020) investigated the photothermal effects of nanocomposites of zeolite imidazole framework-8 encapsulated with humic acid (HA) (ZIF-8) (HuA@ZIF-8) under NIR light for controlled release of Zn^2+^ ions, resulting in a synergistic effect and the ablation of *S. aureus* and *E. coli*. In addition to ZnO and GO-based nanoparticles, the development of semiconductors with a low level of toxicity and highly active nature has emerged as a novel strategy for photothermal antimicrobial activity against *Pseudomonas aeruginosa*, *E. coli* and *S. aureus* (Yougbaré et al., 2020). Semiconductors such as molybdenum disulfide (MoS_2_) have attracted attention for their high band gap and photothermal properties (Gupta et al., 2020). MoS_2_ nanomaterials could result in ROS generation and ablation of bacteria with antioxidant enzyme-like activity (Yu et al., 2021). Studies on chitosan modified MoS_2_ coating loaded with Ag-NPs designed on titanium plates (CS/Ag/MoS_2_-Ti) exhibited both photodynamic and photothermal efficiency under 660 nm visible light, resulting in ROS production and bacteria killing (Zhu et al., 2020). Furthermore, *in vivo* investigations showed the antibacterial efficiency of CS/Ag/MoS_2_-Ti nanomaterials against *S. aureus* and *E. coli* with 98.66% and 99.77% antibacterial activity (Zhu et al., 2020).

Besides, the broad-spectrum antimicrobial properties of nanozymes are gaining attention and are seen as a novel class of therapeutics. Wang et al. (2021) reported the use of Cu single atom sites/N doped porous carbon (Cu SASs/NPC) nanozyme with enhanced photothermal and peroxidase-like activity in the presence of H_2_O_2_ resulting in the generation of •OH radicals to remove the bacterial cell membrane under NIR irradiation. Similarly, the coupling of copper sulfide and lignin as lignin-copper sulfide nanocomposites against *E. coli* and *S. aureus* under NIR light inactivation resulted in synergistic photothermal effects, peroxidase-like activity, and permeabilization of the cell membrane in the presence of H_2_O_2_ (Xie et al., 2021). However, the catalytic effects of nanozymes are reduced in wound infection microenvironments because of the overexpression of GSH levels (as a result of anaerobic glycolysis). However, there have been some recent efforts to overcome these problems and improve the chances of clinical applications of nanozymes. For example, Li Y. et al. (2021) developed an ionic covalent organic framework-based nanozyme that has self-promoting antibacterial potencies with good biocompatibility as a glucose-triggered cascade atalyst against bacterial wound infection. Along with the effective conversion of glucose to H_2_O_2_, gluconic acid produced by providing a compatible catalytic environment improved peroxidase activity to generate more hydroxyl radicals, toxic to bacterial pathogens. Zhang Y. et al. (2021) reported *in vivo* antibacterial activity of oxygen-vacancy molybdenum trioxide nanodots (MoO_3__–X_NDs) that possess photodynamic, photothermal, and nanozyme activity against ESBL *E. coli* and MRSA during NIR irradiation that increased while treating wound healing.

**FIGURE 2:**
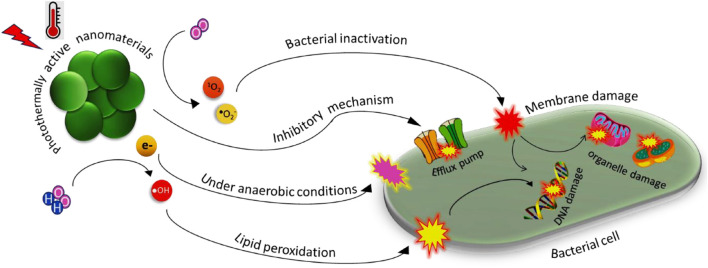
Antibacterial potency of photothermally active nanomaterials via inducing the generation of reactive oxygen species for targeting cell membranes and their permeability.

Furthermore, due to their high biocompatibility and low toxicity levels, quaternary ammonium compounds (QACs) are also being used to keep bacteria heat sensitive and thus cause damage to cell membranes (Jiao et al., 2017). Studies on the synergistic effect of QACs with carbon-based dots such as Cu-RCDs-C35 under 808 nm NIR irradiation resulted in effective killing of *E. coli* and *S. aureus* bacteria through ROS generation (Chu et al., 2021). The *in vivo* anti-infective properties of Cu-RCD-C_35_ were evaluated using the *S. aureus* infected mice model by quantitatively evaluating the efficacy of Cu-RCD-C_35_ bacteria around wounds using lasers that result in bactericidal activities. Figure 2 shows how PANs induce bactericidal effects on pathogens through ROS generation and consequent cell membrane damage.

### Photothermally Active Nanomaterial-Based Bacterial Drug Efflux Pump Inhibitors

The exploration of PANs as bacterial drug efflux pump inhibitors (EPIs) has attracted attention as an effective way to combat drug-resistant bacterial pathogens (Chopra and Dhingra, 2020). EPs facilitate and regulate the efflux of toxic substances, metabolites, and antibiotics from bacterial cells into the outer environment (Shriram et al., 2018; Yu et al., 2020). Recent publications have confirmed the overexpression of multidrug EPs as one of the major resistance mechanisms in bacteria to throw out most antibiotics, including the last line of defense such as colistin (Kim et al., 2020). Reports on the biocompatibility of PANs along with strong EP inhibitory potentials have provided a worthwhile platform for research aimed at addressing the challenges of bacterial drug resistance (Yang et al., 2021). Similarly, EPs have been found to play an important role in biofilm formation that leads to bacterial drug resistance (Alav et al., 2018). Studies have reported that various EPs such as NorA in MRSA (El-Baky et al., 2019), MexAN-OprM in *P. aeruginosa* (Alav et al., 2018), AdeFGH in *Acinetobacter baumannii* (He et al., 2015) are directly involved in biofilm formation. Nano-strategies are also being explored for their antibiofilm potentials by inhibiting these EPs (Dey et al., 2022).

Because of the ferromagnetic properties at ambient temperature, ZnO is considered the choice of nanomaterials by researchers. In addition, the photothermal effectiveness of these oxides can be increased by doping them with metals, resulting in increased magnetic and optical properties (Naveed Ul Haq et al., 2017). Thiolated chitosan-coated cobalt zinc oxide (Co-ZnO) nanomaterials were observed with higher photothermal activity upon sunlight activation, resulting in inhibition of the efflux pump and ROS generation in MRSA (Iqbal et al., 2019). Similarly, studies on methylene blue (MB) photosensitizer and Concanavalin A (ConA) attached dextran capped gold (GNP_DEX_) nanoparticles (MB@GNP_DEX_-ConA) showed EP inhibition and other multi-target modality by singlet oxygen generation and cytotoxicity, resulting in enhanced killing of MDR *Klebsiella* (Khan et al., 2020).

In addition to ZnO and Au NPs, nanoceria, also known as cerium oxide, have the unusual property of flipping their oxidation states due to a high energy band gap with a wide excitation energy and show high catalytic activity (Thakur et al., 2019). Therefore, doping cerium oxide nanomaterials with metal/transition metals led to the generation of surface defects with high-electron-trapping systems that ultimately enhanced the photothermal/photocatalytic activity and further lead to cytotoxicity and ROS production. Furthermore, investigations on thiolated iron-doped nanoceria in visible light photoactivation resulted in ROS production (singlet oxygen and inhibition of hydroxyl radicals) and efflux pump inhibition in pathogens originated from hospital effluents, which revealed its photocatalytic activity against bacterial pathogens with MDR (Khan et al., 2019).

### Photothermally Active Nanomaterial-Based Bacterial Antibiofilm Agents

In therapeutic practice, PTTs have been commonly used to treat biofilm-related infections and develop new technologies to combat drug-resistant bacteria (Tan et al., 2018). Biofilms are primarily defined as complex frameworks, frequently linked with the onset of different infectious diseases that have raised various issues of AMR and HGT. They have emerged as one of the main resistant mechanisms by creating different communities that can adhere to the surface using extra polymeric material such as exopolysaccharide and glycocalyx for their survival in the environment (Sadekuzzaman et al., 2015). The biofilm formation of microorganisms ultimately protects them from harsh environmental and physiological conditions/factors such as dehydration, biocides, etc. (Dunne, 2002). Besides, biofilm formation is influenced by different environmental stress factors. Further, quorum sensing (QS), overexpression of multidrug EPs, and persistence cell formation are the characteristics of the biofilm microenvironment and nanomaterials are finding their use in targeting them for curing bacterial infections (Rao et al., 2021).

**FIGURE 3:**
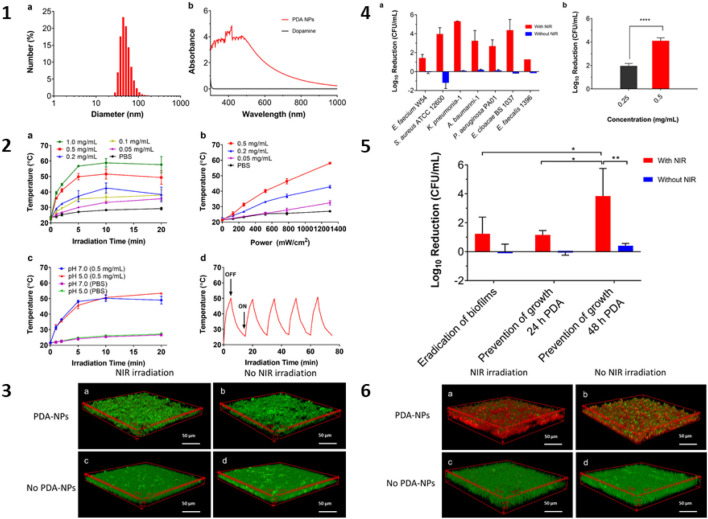
Eradication of ESKAPE panel pathogens biofilms using photothermal polydopamine nanoparticles (PDA-NPs). (1) characterization of PDA-NPs, (2) photothermal properties of PDA-NPs at NIR radiation (880 nm); (3) CLSM image for photothermal killing of PDA-NPs against *S. aureus* biofilms (48 h old) in suspension; (4) killing of ESKAPE-panel pathogens using PDA-NPs (48 h); (5) photothermal killing after NIR-irradiation of biofilms exposed to PDA-NPs; (6) CLSM image of *S. aureus* biofilms after photothermal treatment (48 h). Adapted from Gao et al. (2021). Copyright (2021) Elsevier.

Gold nanocomposites are intensively investigated PTAs for photothermal lysis against several bacterial species. Gold (core) and copper (I, II) sulfide (shell) nanocomposites (Au@Cu_2__–X_S) under NIR irradiation have been investigated with the effect of the decomposition of exopolysaccharides and proteins, primary components of the biofilm that lead to the effective killing of *Enterococcus faecalis* (95%) and *Fusobacterium nucleus* (98%) in biofilm (Cao et al., 2021). Evaluation of further *in vivo* root canal infection models from Beagle dog premolars demonstrated excellent antibacterial activities of Au@Cu_2__–X_S nanomaterial as novel agents with potential clinical applications in root canal therapy (Cao et al., 2021). A photo-illuminated nanostructured TiO_2_ based thin film (NsARC) was used to grow *E. coli*, *S. aureus*, *Saccharomyces cerevisiae*, and *P. aeruginosa* showed antimicrobial activity and biofilm disruption resulting in efficient photocatalytic activity (Wasa et al., 2021). Similar results were observed in cerium oxide nanoparticles doped with tin (Sn-doped CeO_2_) synthesized from *Allophylus cobbe* exhibiting concentration-dependent antibiofilm activity under visible light in *Listeria monocytogenes* and *S. aureus* at 512 mg/mL with a 2 log CFU reduction (Naidi et al., 2021). In addition to Au nanocomposites, plasmonic silver nanocomposite films under NIR irradiation have been studied to photothermally eradicate biofilms in clinically relevant *E. coli* and *S. aureus* (Merkl et al., 2021). Dai et al. (2021) studied nanocomposites based on MSN (FITC Cu_2__–X_SNPs MSNs) as combined NIR-activated photodynamic/PTT for the ablation of biofilms against *P. aeruginosa* and *S. aureus* (90% inhibition rate at a concentration of 160 μg/mL concentration). Furthermore, Zhu et al. (2021) studied biofilm breakdown using a cationic chitosan-coated ruthenium dioxide nanozyme (QCS-RuO_2_@RBT, SRT NS) that exhibited NIR radiation enhanced peroxidase-like activity, resulting in cleavage of extracellular DNA through •OH formation and almost 100% disruption in the biofilm of MDR *P. aeruginosa*. The authors further confirmed the excellent *in vivo* antibacterial activities against chronic *P. aeruginosa* lung infections in the mouse model (Zhu et al., 2021). In another interesting study, Hu et al. (2017) developed surface-adaptive gold nanoparticles (Au-NPs) that exhibited a self-adaptive target to the acidic microenvironment of the MRSA biofilm, which was ablated by nanoparticles under NIR light irradiations; in particular, no damage was observed to the healthy tissues around the biofilm. Further *in vivo* studies on bactericidal effects in rabbit models infected with MRSA resulted in photothermal killing of bacteria (Hu et al., 2017). MRSA biofilms are the main bacterial infections that are involved in bone implant failure (Khatoon et al., 2018; Li Y. et al., 2020). Recent studies on the elimination of MRSA biofilm infection in bone implant using polyvinyl alcohol modified with chitosan, polydopamine and NO release donor in the red phosphorous nanofilm deposited in titanium implant (Ti-RP/PCP/RSNO) under NIR light irradiation resulted in the formation of peroxynitrite (•ONOO^–^) that affected the gene regulation of biofilm formation factors, i.e., staphylococcal accessory regulator (SarA), intercellular adhesion gene A (icaA), and intercellular adhesion gene D (icaD); and virulence factors, i.e., staphylococcal enterotoxin A (SEA) and α-hemolysis resulting in inhibition of biofilm formation and damaging MRSA (Li Y. et al., 2020). *In vivo* studies further confirmed excellent osteogenesis and biofilm eradication by NO released from the RP/PCP/RSNO system under NIR irradiation, indicating non-invasive tissue reconstruction of MRSA-infected tissues through phototherapy and immunotherapy (Li Y. et al., 2020). Similar *in vivo* studies for the elimination of MRSA biofilms and excess inflammation from severely infected wound models of abscess using deoxyribonuclease (DNase)–carbon monoxide (CO)@mesoporous polydopamine nanoparticles (MPDA NPs) under NIR irradiation and resulted in biofilm extracellular DNA degradation by DNase I and their destruction by released CO gas molecules leading to synergistic PTT (Yuan et al., 2021). The anti-inflammatory properties of the released CO resulted in alleviation of the inflammatory response, therefore accelerating the wound healing process and showing their potential therapeutic applications in the MRSA biofilm caused clinical problems (Yuan et al., 2021). Figure 3 illustrates the eradication of pathogens from the ESKAPE group that form biofilms using polydopamine nanoparticles (PDA-NPs) under NIR irradiation. In conclusion, though there is considerable progress in laboratory-based *in vitro* and *in vivo* studies for establishing the antibacterial potencies in general and antibiofilm activities, in particular of PAN-based antibiofilm agents, there is not much translational success, and there is much scope for improvement in their journey from the laboratory to the bedside.

**FIGURE 4:**
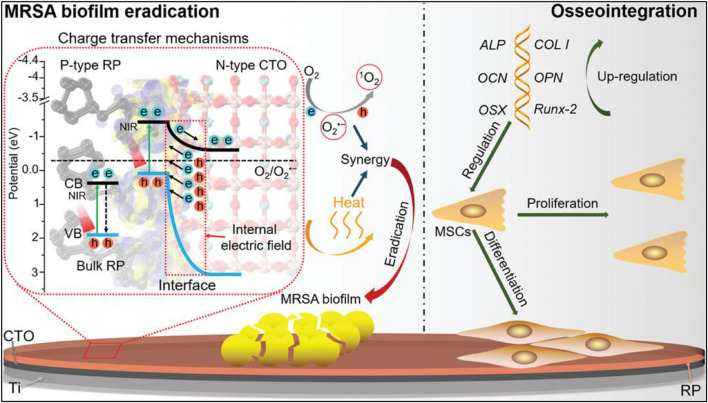
Mechanism for photothermal and Photocatalytic Eradication of MRSA Biofilms and Osseointegration Using Oxide Perovskite-Based P–N Heterojunction under Enhanced Near–Infrared (p-type CTO, perovskite-type calcium titanate; p-type RP, perovskite-type red phosphorous; ALP, alkaline phosphatase; OCN, Osteocalcin; OPN, Osteopontin; OSN, Osterix; RunX-2, runt-related transcription factor 2; COL I, Collagen type I; MSCs, mesenchymal stem cells; CB, conduction band; VB, valence band). Adapted from Mao et al. (2021). Copyright (2021) Wiley-VCH.

Spurred by the successful photothermal bactericidal applications of Au, Ti, and Ce-based nanocomposites, nanoswimmers are being explored for *in vivo* deep-layer biofilm elimination considering their excellent self-propelling and penetrating abilities (Cui et al., 2020). The NIR light-driven nanoswimmer was found to be efficient with self-propulsion, penetrating, and thermal-triggered release of the vancomycin (Van) antibiotic as chemo/PTT, resulting in *in vivo* biofilm elimination and the treatment of biofilm-associated infections (Cui et al., 2020). Figure 4 shows the mechanisms for the photothermal and photocatalytic eradication of MRSA biofilms.

### Photothermally Active Nanomaterial-Based Bacterial Anti-quorum Sensing Agents

Quorum sensing is characterized as microbial communication systems driven by various compounds or auto-inducers mediated through bacterial metabolic pathways. Various molecular mechanisms of QS systems such as acyl-homoserine lactones, peptide auto-inducers, and auto-inducer 2 are adopted by microbes for intercellular signaling (Irie and Parsek, 2008). As a result, there is a growing desire for commercially feasible, innocuous/anti-QS agents that have parallel action modes that address both antibiofilm formation and anti-QS. QS inhibitors (QSI), including nanomaterials and photothermal therapeutics, have been reported with potential to disrupt the QS system and inhibit biofilm formation (Geske et al., 2005). In recent years, PANs have been reported as effective agents for eliminating microbial QS systems. Although this area is far from its full potential exploration and requires more and comprehensive investigation, however, the recent trends are encouraging.

**FIGURE 5:**
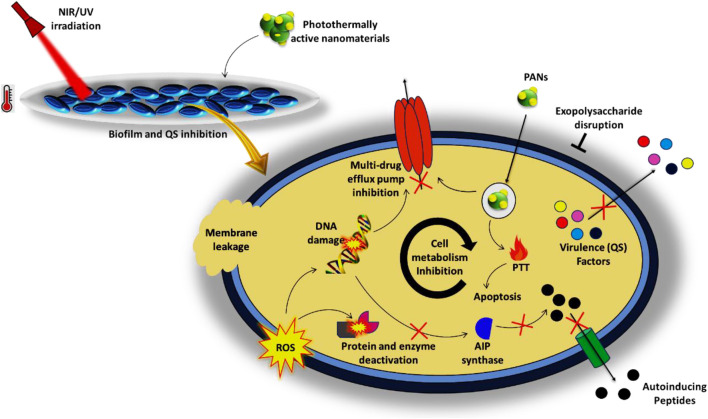
Antibiofilm and quorum-sensing inhibitory potencies of photothermally active nanomaterials.

Sun et al. (2020) developed a synergistic antibiofilm system combining QSI, antibiotic, and PDT in multidrug-delivering hollow carbon nitride spheres (HCNSs), which successfully inhibited the QS system by influencing the production of virulence factors and surface hydrophobicity leading to a reduction in biofilm formation to 10.3%, even lower than the projected additive value (37.4%, ^∗∗^*p* = 0.002). In addition, *in vivo* studies were carried out to evaluate the cytotoxicity and efficacy of the nanosystem in MRSA biofilm implanted periprosthetic infection (PPI) mice model, which resulted in the killing of bacteria and regeneration of the epidermis and wound healing (Sun et al., 2020). Similarly, investigations in NIR light-enhanced protease-conjugated gold nanorods (200 μg/mL) revealed their anti-QS ability by eliminating exotoxins (86%) and biofilm in *E. coli* (70.5%) and *S. aureus* (93.3%) (Li et al., 2019c). Figure 5 illustrates the antibiofilm and anti-QS potencies of PANs.

## Photothermally Active Nanomaterials as Antibacterial Drug Carriers

Inadequate drug concentration at the target infection site, drug degradation before reaching the target, increase in frequency of drug administration, low bioavailability, limited penetration at the infection site, increased side effects associated with higher drug dosage are some of the common problems known to compromise the efficacy of antibiotics and ultimately can lead to the increase in drug resistance of pathogens (Kalhapure et al., 2015; Canaparo et al., 2019; Li Z. et al., 2021). To overcome some of these problems, effective and safer drug delivery systems comprising PANs that encapsulate or conjugate antibacterial drug(s) have been explored in recent years (Table 2). Au-NPs, Ag-NPs, GO nanomaterials, FeO nanomaterials, and black phosphorus are some of the nanomaterials with photothermal conversion efficiencies under NIR irradiation that have been significantly utilized for rapid antibacterial drug delivery at the target infection site. The synergistic combination of antibiotics and PANs as drug carriers enhances antibacterial activity and also reduces toxic side effects; for example, effective eradication of drug resistant bacteria through combined photothermal chemotherapy has been studied using a biocompatible thermo-responsive inspired drug delivery nanotransporter (TRIDENT) (Qing et al., 2019). The TRIDENT nanosphere encapsulating imipenem and the photosensitizer molecule IR780 (IMP/IR780@TRN) releases imipenem at the target site via the phase transition generated by the temperature induced by NIR and damages the bacterial cell membrane, facilitating imipenem permeation and eventual bacterial death with the log_10_ (CFU mL^–1^) value reduced to 4.23 ± 0.01 for *E. coli* and 4.14 ± 0.02 for *S. aureus* (Qing et al., 2019). With a further increase in body weight, a reduction in the size of the skin lesion (by approximately 70% for MDREC and 60% for MRSA) was observed in a mouse model of skin infected with MDR *E. coli* and MRSA. Another study developed an NIR light-driven nano swimmer (HSMV) that effectively combines PTT and chemotherapy in one system that performs efficient self-propulsion and penetrates deep into the bacterial biofilm, increasing the photothermal removal of *S. aureus* (>90%) and the killing of bacterial cells plus a 5-fold log reduction (∼99.99%) in biofilm colonies to eradicate bacterial biofilms (Cui et al., 2020). Furthermore, *in vivo* studies showed antibiofilm efficacy in mouse implant-related PPI model with complete wound healing and negligible damage to major organs such as heart, spleen, liver, kidney, and lungs, indicating good therapeutic biosafety of HSMV (Cui et al., 2020).

**TABLE 2:** Examples of photothermally active nanomaterials as drug carriers.

Nanomaterial	Antimicrobial drug encapsulated	Light irradiation	Targeted bacteria	An advantage provided by nanomaterials	References
Gold nanoparticle	Vancomycin	Near-infrared (NIR) light irradiation (808 nm for 5 min)	*Staphylococcus aureus*, *Streptococcus pyogenes*, *Escherichia coli* 0157:H7, *Acinetobacter baumannii*, vancomycin-resistant *Enterococci* (VRE), methicillin-resistant *Staphylococcus aureus* (MRSA), pandrug-resistant *A. baumannii*	Effective killing of pathogenic bacteria through selective recognition of the bacterial cell wall by vancomycin, less than 1% survival fraction achieved through hyperthermia, reduction in the time required to inhibit pathogenic growth compared to antibiotics, which generally requires 1–2 weeks.	Huang et al., 2007
AuNC@PDA	Daptomycin	Diode Laser irradiation (808 nm for 10 min)	*S. aureus*	Selective delivery of nanoconstruct directly onto the bacterial cell and high efficacy against bacterial biofilm.	Meeker et al., 2016
HSKAu_rod_	Kanamycin	Near-infrared (NIR) light irradiation (780 nm for 20 min)	*E. coli* BL21	Synergistic antibacterial activity attributed to local thermal ablation provided by AU nanorods under NIR irradiation, increased penetration of kanamycin inside bacteria, inhibition of protein synthesis by binding to the 30S ribosomal subunit.	Hu et al., 2013
PDA NP-Cip/GC hydrogel	Ciprofloxacin	Near-infrared (NIR) light irradiation (808 nm for 10 min followed by an interval of 30 min)	*S. aureus*	Effective inactivation of *S. aureus* accompanied by a synergistic effect between local hyperthermia and controlled release of ciprofloxacin in target infection, inactivation of gyrase and topoisomerase IV enzymes inhibiting DNA synthesis.	Gao et al., 2019
DAP-GCS-PDA@GNRs	Daptomycin	Near-Infrared (NIR) laser irradiation (808 nm for 7 min)	Methicillin-resistant *S. aureus*, vancomycin-resistant *Enterococci*	Acidity-activated release of DAP effectively damages the bacterial cell membrane, causing increased permeability and reduction of heat resistance of the cell membrane, increased drug delivery at the target infection site by hyperthermia, negligible biotoxicity, and increased wound healing both *in vivo* and *in vitro.*	He et al., 2018
GelMA-Au NBPs@SiO_2_ hybrid hydrogel	Minocycline	Near-infrared (NIR) light irradiation (808 nm for 1, 3, 5 min)	*Porphyromonas gingivalis*	Controlled release of minocycline and lower retention of bacterial retention after treatment.	Lin et al., 2020
Van-LaB_6_@SiO_2_/Fe_3_O_4_	Vancomycin	Near-infrared (NIR) light irradiation (808 nm for 5 min)	*S. aureus*, *E. coli*	The survival fraction of *S. aureus* and *E. coli was* reduced to less than 1% because the magnetic property of nanomaterials was used that increased the efficacy of the nanomaterial for the capture and photothermal ablation of *S. aureus* and *E. coli*	Lai and Chen, 2013
Au@Van NPs	Vancomycin	Near-infrared laser irradiation (808 nm within 5 min)	Vancomycin-resistant *Enterococci*	Vancomycin combined with gold nanoparticles showed a 16-fold reduction in vancomycin concentration required for VRE treatment compared to the free form of vancomycin	Wang S. G. et al., 2018

In recent years, reduced accumulation of antibiotics in healthy tissues and prevention of exposure of the commensal microflora to a sublethal dose of antibiotics are the main challenges faced in the development of effective drug delivery systems. In an interesting attempt, vancomycin and thiol-poly(ethylene glycol) (mPEG-SH) molecules modified polydopamine nanoparticles (PDA-PEG-Van) exhibiting photothermal activity with long-term stability and specific MRSA target ability resulted in a multivalent hydrogen bond interaction between vancomycin and the MRSA cell wall to treat MRSA bacterial infections and further prevention of damage to healthy tissue (Hu et al., 2019). Similarly, Zhao et al. (2019) developed a nanotherapeutic decorated with a bioinspired heteromultivalent ligand that specifically recognized *P. aeruginosa* by a glycomimetic shell that protects native cells from infection in addition to bacterial biofilm prevention (rate of up to 85%) and biofilm dispersal by more than 80% with severely damaged ruptured cell surface. Finally, the *in vivo* study evaluated the efficacy of nanotherapeutic against acute pneumonia reporting bacterial clearance from the lungs (*p* < 0.05), downregulated amount of inflammatory cells (neutrophils, lymphocytes, and macrophages) and decreased cytokine levels (*p* < 0.05), effectively reducing the immune response and eliminating acute bacterial pneumonia (Zhao et al., 2019). Reports on an injectable system of hollow microspheres that is effective in treating subcutaneous abscesses under NIR irradiation demonstrated an increase in the cytotoxicity of bacteria in the abscesses and spatial stabilization of encapsulated vancomycin at the injection site (Chiang et al., 2015).

Other antimicrobial drugs, such as antimicrobial peptides (AMPs), enzymes, and plant products, have also been identified to be administered via PANs, expanding the number of therapy options. The development of amino-modified mesoporous silica-coated gold nanorods encapsulating cinnamaldehyde (CA@AuMN-HA) under NIR irradiation showed strong antibacterial activity with controlled CA released property and up to 90% inhibition when MRSA was incubated with CA@AuMN-HA (80 μg/mL) and exposed to an 808 nm laser (Sun et al., 2018). A further study on cytotoxicity and histological analysis demonstrated a slight influence on cell viability at a high concentration of 320 μg/mL with no histological abnormalities or organ damage, concluding excellent biocompatibility and non-toxicity of the synergistic system for clinical application. Furthermore, Moorcroft et al. (2020) designed a nanoparticle-loaded hydrogel consisting of AMP IK8-loaded liposome incorporated into polyethylene glycol (PEG) along with gold nanorods under laser irradiation that led to photothermally triggered release of IK8 and enhanced AMP efficacy in *P. aeruginosa* and *S. aureus*. Despite the potential of AMPs as an antibiotic alternative, clinical translation is still greatly hindered by proteolytic instability *in vivo*. Therefore, the encapsulation of IK8 within the lipid bilayer restricts the access of protease by providing a protective barrier and, therefore, can facilitate the treatment of an infectious environment rich in proteolytic enzymes, such as open wounds. Henceforth, considering the advantages of PANs, including high drug bioavailability, no or lesser toxicity, and efficient delivery of antibiotics in healthy host tissue, the PANs present tremendous therapeutic potential as antimicrobial drug carriers.

One of the important factors in developing nanoparticle-based drug delivery is the high drug loading since most of the nanocarriers have low drug loading which led to the use of a large amount of nanomaterial causing adverse side effects and increased in the cost of nanotherapeutics (Della Rocca et al., 2012). Therefore, to alleviate these limitations, PANs with high loading capacity are being successfully developed, for example, a hydrogel/liposome system containing IK8 loaded liposome and lipid-coated nanorods that allowed the encapsulation of IK8 (770 ± 21 μg mL^–1^) against *S. aureus* (32 μg/mL) (Moorcroft et al., 2020). Another study reported that polydopamine (PDA) coated gold nanorods (GNRs) grafted with glycol chitosan (GCS), i.e., GCS-PDA@GNRs showed a high loading capacity of daptomycin (DAP) in GCS-PDA@GNRs (552.32 μg of DAP at a 0.2 mg/mL of DAP concentration) (He et al., 2018). In addition to high drug loading capacity, PANs can also load two drugs simultaneously, thus enhancing the efficacy of treatment, for example, Sun Y. et al. (2019) developed HCNS/A&L@HA nanomaterial where luteolin (L) as a QSI and ampicillin (A) antibiotic were encapsulated in HCNSs capped by hyaluronic acid (HA) showed sequenced release of luteolin (first release) and ampicillin (second released) with biofilm inhibition efficiency of 64.2%. Similarly, PANs were also reported to be capable of loading plant essential oils, known for their striking antimicrobial properties (Sun et al., 2018). These case studies highlight the high loading capacity and drug loading diversity as advantages of PANs over other nanomaterials.

However, despite these advantages, some limitations still exist with the PAN application that need to be further addressed. These include drug loading efficiency; accurate antibacterial mechanism identification; difficulty in killing heat resistant bacteria when treated at high temperatures such as 45°C, and poor tissue penetration by a light source limiting the clinical application of PANs. Therefore, the development of photosensitizers based on the second NIR window (NIR-II, 1000–1700 nm) is seen to be the focus of future research in this area (Wei et al., 2020).

## Potential Cytotoxicities Associated With Photothermally Active Nanomaterials and Strategies to Minimize Them

Foreign matter, especially at the nanoscale, needs to be thoroughly and carefully examined for potential cytotoxicities prior to its clinical applications. Furthermore, they must be investigated to minimize their impacts on normal (or non-target) cells. One of the greatest concerns associated with the therapeutic/clinical use of PANs is that they can easily pass through cells and tissues and could enter the systemic circulation. Chen et al. (2019) highlighted some of the issues related to the surface effect of nanomaterials that can easily be changed by ROS to become toxic and damaging to bacterial cells. However, for better antibacterial applications of PTT, an extensive understanding of PTA and potential toxicities/side effects is required (Chen et al., 2020). In one such attempt, studies on the cytotoxicity of photothermally active copper sulfide (CuS) nanoplates demonstrated that cell viability decreased in human umbilical vein endothelial cells (HUVECs) and mouse leukemic monocyte macrophage cells (RAW 264.7) when treated with higher concentrations (>100 μg/mL) (Feng et al., 2015). During the photothermal effect of nanomaterials, heat dissipation in the energy loss pathway occurs after the light is absorbed by the nanoparticles; therefore, the maintenance of uniform heating of nanoparticles is highly recommended for the uniformity of the reaction. To obtain controlled photothermal responses, it is crucial to acquire nanoparticles with low heterogenicity in size and shape, narrow absorbance line width, and high extinction. Nanoparticles with established pharmacokinetic aspects (absorption, distribution, metabolism, and excretion) are needed to minimize dosage to avoid unwanted side effects or toxicity (Kim et al., 2019). Some approaches to minimize the side effects of PANs are discussed below.

### Combining Photothermal Therapy With Other Therapies

The use of high temperature in PTT can be painful to the patient and can cause injuries to nearby tissues. Hence, to minimize this side effect and maximize antibacterial efficiency, lowering the temperature can significantly help. Therefore, the combination of PTT with PDT (PTDT) is an emerging synergistic therapeutic approach (Sun J. et al., 2019). PDT is dependent on the photosensitizer (PS) that produces ROS and causes the death of bacteria by destroying cellular DNA, lipids, and proteins. An increase in temperature results in an enhancement of the bacterial cell membrane, facilitating the uptake of PS and PDT agents (Sahu et al., 2016). The development of a single NIR laser-excited PTDT system resulted in the generation of low temperature, resulting in the killing of bacteria and ROS production with a 95% antibacterial rate in *S. aureus* and *E. coli* (Sahu et al., 2016). As PDT is limited by oxygen concentration, to combat the biofilm microenvironment, non-catalytic antibacterial therapy is used as an alternative to PDT with synergistic therapy (de Melo et al., 2021). A combination of a PDA@Au-hydroxyapatite hybrid system with a low concentration of H_2_O_2_ showed enhanced antibacterial activity for *E. coli* (96%) and *S. aureus* (95.2%) resulting in a significant increase in wound healing (Xu et al., 2018). Li et al. (2019d) developed a biocompatible phototherapeutic system composed of MoS_2_, IR780 photosensitizer, and arginine–glycine–aspartic acid–cysteine (RGDC), where magnetron-sputtered MoS_2_ showed photothermal property plus IR780 that produced ROS with NIR irradiation (λ = 700–1100 nm) photodynamic property together with the assistance of glutathione oxidation that provided synergistic and rapid killing of bacteria 98.99 ± 0.42% eradication ratio against a biofilm of *S. aureus*. Synergistic killing of bacteria by combining the photodynamic activity of GO and the photocatalytic activity of Ag-NPs (GO/Ag/collagen coating) under laser irradiation resulted in high bacterial efficiency against *E. coli* (96.3%) and *S. aureus* (99.4%) reported by Xie et al. (2017). Li J. et al. (2021) Designed nanoparticles embedded at the semiconductor metal interface called the interfacial Schottky junction of bismuth sulfide (Bi_2_S_3_) and titanium carbide (Ti_3_C_2_T_x_) nanosheets that exhibited photocatalytic activity resulting in the eradication of *S. aureus* (99.86%) and *E. coli* (99.92%) under NIR irradiation.

### Bacterial-Targeted Photothermal Therapy

Direct bacterial targets are one of the effective approaches to combat bacterial infections that help reduce side effects and enhance therapeutic efficiency (Huang et al., 2019). Alhmoud et al. (2017) used *S. aureus* targeting antibodies to increase efficiency and reduce side effects, resulting in reduced cell viability (99.99%) and increased antibacterial efficiency (up to 10 times). However, because of drawbacks such as instability, rapid decay, complex reaction process, and high price, the use of other molecules, such as small-size DNA/RNA aptamers, took over because of their negligible immunogenicity, high loading capacity, easy synthesis, and mainly great stability. Ocsoy et al. (2017) synthesized gold nanorods decorated with DNA aptamer showed great photothermal anti-MRSA activity with 95% bacterial inactivation. However, these strategies are effective only when the pathogen is known; therefore, to target unknown bacteria, the use of cationic molecules emerged as another potential targeting strategy. Fan et al. (2019) used cationic Magainin I (Mag I) as magainin-modified polydopamine nanoparticles to improve PDA affinity. The cell-derived membrane has many advantages in its use *in vitro* as a coating material for targeted therapy. Wang C. et al. (2018) used the extrusion method to coat Au-Ag nanocages with *S. aureus* pretreated macrophage membranes. The American national standards stated that the laser range for *in vitro* use, maximal penetration depth, and maximum permissible exposure for skin is 1000–1100 nm and 1 W/cm^2^. Compared with the continuous mode of laser, the effective strategy to be found was the pulsed laser with a short duration of time with less heat diffusion and specific killing of bacteria. Kirui et al. (2019) and Yuan et al. (2015) used a nanosecond pulsed laser and a femtosecond pulsed laser, respectively, and showed that antibacterial PTT did not cause obvious cytotoxicity *in vivo*. The new trend of using non-chemical antibacterial means for the disinfection of environments is grabbing the attention. Wu et al. (2021) developed safe and biodegradable photosensitive red phosphorus nanoparticles (RNPs) that exhibit strong photothermal and photocatalytic ability that resulted in the death of *S. aureus* (99.98%). Investigations on silver sulfide (Ag_2_S) nanoparticles decorated nanocubes (Ag_2_S/NCs) exhibiting excellent photothermal properties and biocompatibility resulted in increased antibacterial efficiency against *S. aureus* (97.3%) under light irradiation (Xiong et al., 2019). Li et al. (2019b) studied the synergistic effect of lysozyme-assisted anti-infection and photothermal eradication of *in vivo* MRSA using naturally derived biodegradable human hair melanosome nanostructures resulting in highly efficient antibacterial activity (97.19%) and tissue reconstruction, thus accelerating tissue repair. Mao et al. (2018) reported repeatable killing of bacteria (*E. coli* 98.90% and *S. aureus* 99.51%) through fabricated hybrid hydrogen gel embedded with 2D few-layer black phosphorus nanosheets (BP) via electrostatic interaction.

### Controlled Release of Photothermal Agents

The microenvironment surrounding the bacteria is often pH dependent, due to the release of various enzymes and products into the surroundings. Zhang et al. (2019) synthesized Rhenium trioxide (ReO_3_) nanocubes were effective in the acidic infectious environment, however, rapidly degraded in healthy tissues. In addition, toxins and enzymes also showed activity to trigger and release PTAs. Similarly, Yang et al. (2017) developed a supramolecular complex that reduces radicle anions on the bacterial surface such as *E. coli*. Specific PTT was achieved by the photothermal effects of radicle anion under NIR irradiation. Xie et al. (2018) designed a hybrid polydopamine (PDA)/Ag_3_PO_4_/GO coating and observed that controlled release and photocatalytic Ag_3_PO_4_ produce synergistic antimicrobial efficiency up to 99.53 and 99.66% against *E. coli* and *S. aureus*, respectively. Other approaches such as bacterial capture and killing are also used as alternative strategies. Studies have reported that prussian blue nanoparticles synthesized as photosensitive hydrogels exhibiting strong photothermal properties under NIR irradiation resulted in the capture of bacteria in hydrogels and its further killing by perturbation of bacterial cell membranes in *S. aureus* (99.97%) and *E. coli* (99.93%) (Han et al., 2020b). Similar results were observed in MRSA-infected osteomyelitis to treat tissue infections using magnetically iron oxide-targeted carbon nanotubes (Fe_2_O_3_/CNT), resulting in microwave-assisted bacterial killing (Qiao et al., 2020). However, more studies are needed to further reduce the cytotoxicities of PANs.

## Conclusion and Future Outlook

In the current review, we have meticulously reviewed the literature and summarized the ongoing advances in the photothermal inactivation of drug-resistant bacterial pathogens using nanoparticles under broad-spectrum irradiation, i.e., ultraviolet, visible, and infrared light, with a potential approach for addressing the AMR problems. Several nanomaterials, especially metal and metal oxide bases, are known for their antibacterial potencies; however, their associated toxicities, potential side effects to non-target cells, cost, and lesser biocompatibility have limited their therapeutic applications. To overcome these limitations, PANs are emerging as potential alternative approaches due to their striking antibacterial potencies with less or no toxicities for the host and biocompatibilities. These nanomaterials have many advantages for generating fast and efficient localized temperatures to eradicate drug-resistant bacterial strains. These nanoagents absorb NIR and UV light for the lysis of bacteria and their ultimate killing via targeting the drug resistance determinants. They target the cell membranes of bacteria by hyperthermia and ROS production in the presence of H_2_O_2_ and O_2_, leading to DNA damage and other protein inactivation. Efflux pumps are also among the main targets that are directly targeted by PANs. PANs have also found their effective use for inhibition of biofilms and QS by influencing bacterial virulence factors and autoinducing factors (AIPs). In addition, the combination of PANs with antibiotics and other materials such as nanocomposites is also being explored for enhanced photothermal effect for bacterial ablation. Interestingly, PANs have also been shown to be potent antibacterial drug carriers with controlled release and target efficiencies. Thus, PANs are of great significance in turning into wide-spectrum antibacterial therapeutics that can combat threatening AMR and contain superbugs. However, more comprehensive investigations are needed to identify new PANs with striking antibacterial potency (both bactericidal and synergistic) with less or no toxic impact on hosts, and such investigations should be undertaken. Studies are needed to decipher the underlying mechanism of action targeted by PANs for effective bactericidal and drug resistance reversal activities. One more issue to be taken into account is the need for higher cell/tissue penetration by the PANs, and thus should be the focus of future research. All these and other pharmacokinetic and pharmacodynamic studies are needed prior to clinical applications and translational success of PANs as effective antibacterial agents against drug-resistant pathogens.

## Author Contributions

VK and JP conceived the idea, wrote and revised the manuscript, and supervised the manuscript. KK, SR, PB, VS, and UA contributed to the literature survey, wrote the manuscript, and drew the figures and tables. JP performed the project administration and funded acquisition. All authors made a substantial contribution to the manuscript revision and approved it for publication.

## Conflict of Interest

The authors declare that the research was conducted in the absence of any commercial or financial relationships that could be construed as a potential conflict of interest.

## Publisher’s Note

All claims expressed in this article are solely those of the authors and do not necessarily represent those of their affiliated organizations, or those of the publisher, the editors and the reviewers. Any product that may be evaluated in this article, or claim that may be made by its manufacturer, is not guaranteed or endorsed by the publisher.
